# Scientific Evaluation of Edible Fruits and Spices Used for the Treatment of Peptic Ulcer in Traditional Iranian Medicine

**DOI:** 10.1155/2013/136932

**Published:** 2013-08-26

**Authors:** Mohammad Hosein Farzaei, Mohammad Reza Shams-Ardekani, Zahra Abbasabadi, Roja Rahimi

**Affiliations:** ^1^Department of Traditional Pharmacy, Faculty of Traditional Medicine, Tehran University of Medical Sciences, Tehran 1417653761, Iran; ^2^Department of Pharmacognosy, Faculty of Pharmacy, Tehran University of Medical Sciences, Tehran 1417614411, Iran; ^3^Faculty of Pharmacy, Kermanshah University of Medical Sciences, Kermanshah 6734667149, Iran

## Abstract

In traditional Iranian medicine (TIM), several edible fruits and spices are thought to have protective and healing effects on peptic ulcer (PU). The present study was conducted to verify anti-PU activity of these remedies. For this purpose, edible fruits and spices proposed for the management of PU in TIM were collected from TIM sources, and they were searched in modern medical databases to find studies that confirmed their efficacy. Findings from modern investigations support the claims of TIM about the efficacy of many fruits and spices in PU. The fruit of *Phyllanthus emblica* as a beneficial remedy for PU in TIM has been demonstrated to have antioxidant, wound healing, angiogenic, anti-*H. pylori*, cytoprotective, antisecretory, and anti-inflammatory properties. The fruit of *Vitis vinifera* has been found to be anti-*H. pylori*, anti-inflammatory, wound healing, angiogenic, cytoprotective, and antioxidant. The fruit and aril of seed from *Myristica fragrans* exert their beneficial effects in PU by increasing prostaglandin, modulation of nitric oxide and inflammatory mediators, wound healing, antisecretory, antacid, antioxidant, and anti-*H. pylori* activities, and improving angiogenesis. Pharmacological and clinical studies for evaluation of efficacy of all TIM fruits and spices in PU and their possible mechanisms of action are recommended.

## 1. Introduction

Gastric and duodenal ulcers, entitled as peptic ulcer (PU), are the most prevalent gastrointestinal disorders in the world [1]. PU is a multifactorial and complex disease with unclear etiological factor. It has been demonstrated that PU is a pathological condition in which biological balance between aggressive and defense factors is disturbed. Among aggressive factors, it can be named from gastric acid and pepsin secretion, active free radicals and oxidants, leukotrienes, endothelins, and exogenous factors such as ethanol or nonsteroidal anti-inflammatory drugs (NSAIDs). In contrast, gastric mucus, bicarbonate, normal blood flow, prostaglandin (PG), nitric oxide (NO), and antioxidant enzymes such as catalase and glutathione (GSH) work as defense factors [2, 3]. Most of the gastric lesions originate from a chronic infection of gastric mucosa with *Helicobacter pylori* (*H. pylori*). *H. pylori* is a common human pathogen with asymptomatic stomach colonization in nearly 70% of the population and approximately 10%–20% are susceptible for PU [4].

Traditional medicines of all over the world possess different virgin remedies for the treatment of symptomatologies related to many ailments. Thus, they are very important for investigation on their efficacy and phytochemical constituents [5–7]. There are several edible fruits and spices proposed in traditional Iranian medicine (TIM) for the management of PU [8, 9]. Present study conducted to review these fruits and spices and found evidence for their efficacy and biological mechanisms in modern publications. In order to achieve this aim, electronic databases including PubMed, Scopus, Web of Science, and Google Scholar were explored for each of the medicinal plants recommended in TIM for PU, and all retrieved articles were evaluated to obtain any *in vitro*, *in vivo*, or clinical evidence for their efficacy and possible mechanisms. The retrieved studies either explain clearly effectiveness of these herbs or indirectly their efficacy on the involved mechanisms in the treatment of PU.

## 2. Edible Fruits and Spices for the Treatment of PU in TIM

Scientific, common English and traditional Iranian names of edible fruits and spices used in TIM for the management of PU with their plant family and pharmacological activities in TIM have been shown in Table 1. Moreover, details of *in vitro* and *in vivo* findings that support their efficacy in PU have been demonstrated in Table 2. Below, these fruits and spices with their possible mechanisms of action in PU have been described alphabetically.

### 2.1. *Amygdalus communis*

*A. communis* demonstrated a trivial antacid property *in vitro* [10], and different parts of fruit showed antioxidant activity [11, 12]. Topical application of bitter almond oil healed wounds in rats [13]. Amygdalin, a glycoside isolated from *Amygdalus* genus, revealed gastroprotective properties through suppression of inflammatory cytokines [14]. 

### 2.2. *Berberis vulgaris* L.

Fruit has shown antioxidant activity [15] and may have a role in improvement of intestinal mucosal morphology [18]. Berberine, as an active constituent of fruit, promoted releasing NO in the intestinal endothelium [16]. It showed inhibitory activity on *H. pylori* growth [17] and gastroprotective effect through modulating NO synthase (NOS) gene expression [19]. It also had protective activity against small intestinal injury and increased adenosine in the intestinal tissue [20]. However, there is a report about dose-dependent gastric ulcer inducing activity of berberine from *B. crataegina* during acute toxicity test in mice [21]. 

### 2.3. *Cornus mas* L.

The fruits showed antioxidant activity [22]. The leaves of *C. controversa, C. macrophylla,* and *C. walteri *demonstrated anti-*H. pylori* activity [23].

### 2.4. *Cucurbita maxima* Duch. and *C. Pepo* L.

Fruit pulp of *C. pepo *showed protective activity against gastric and duodenal ulcer via enhancing mucosal thickness and increasing alkaline phosphatase enzyme in stomach and duodenum tissue [25]. Triterpenoids from the seeds of *C. pepo* protected against gastric ulcer via reducing gastric secretion and free and total acidity of gastric juice and its antioxidant activity [24]. 

### 2.5. *Cydonia vulgaris* Pers. syn. *C. oblonga* Mill

Various components from peel, pulp, and seed of fruit exhibited antioxidant activity [26]. Phenolic compounds from fruits showed gastroprotective properties [27]. Fruit juice and fruit extract demonstrated strong and weak anti-*H. pylori* activity, respectively [29]. The seed mucilage topically administrated heals toxin-induced skin lesions in rabbits [28]. 

### 2.6. *Malus domestica* Baumg

*M. domestica *fruit and its isolated phenolic acids demonstrated gastroprotective activity via reducing neutrophil infiltration in gastric tissue and antioxidant activity [30, 31]. The fruit also reduced gastric endothelial cell injury through antioxidant activity [31]. *M. domestica *peel showed both *in vitro* and *in vivo* anti *H. pylori* activity [32, 33]. Fruit polyphenol revealed gastroprotective activity without significant effect on gastric secretion. It also inhibited lipid peroxidation and production of inflammatory cytokines [34]. However, there is a report on exacerbation of gastric ulcer by fruit polyphenol extract [27].

### 2.7. *Morus alba* L. and *M. nigra* L.

Cyanidin-3-O-glucoside, a component isolated from *M. alba *fruit, showed protective activity against endothelial dysfunction [36]. Ethyl acetate soluble fraction of fruit attenuated gastric ulceration in rat via its antioxidant activity [38]. The leaf had protective activity against gastric ulcer [35] and revealed anti *H. pylori* as well as antioxidant activity [37]. 

### 2.8. *Myristica fragrans* Houtt

Various investigations have proved strong anti-*H. Pylori* activity of* M. fragrans* seed *in vitro* [39]. Dihydroguaiaretic acid isolated from aril of the seed also demonstrated strong anti *H. pylori* activity [40]. The seeds suppressed free and total acidity and volume of gastric secretion [42]. The aril of seed showed antioxidant activity *in vitro* [41]. *M. malabarica* fruits improved gastric ulcer in mice via increasing PG E2 synthesis, improving angiogenesis, modulating NOS gene expression, producing balance between proinflammatory and anti-inflammatory cytokines, and improving mucin content and antioxidant activity in gastric tissue [44–46]. *M. andamanica* leaves demonstrated wound healing activity *in vivo* [47]. A polyherbal formulation containing *M. fragrans *fruits inhibits gastric ulcer and hypersecretion in rats [43].

### 2.9. *Oryza sativa* L.

*O. sativa* bran oil protected gastric mucus from stress-induced ulcers in rats via inhibiting acid secretion. *O. sativa *reduced basal acid secretion and stimulated gastric acid secretion by histamine in rats [50]. Antioxidant activity of normal and pigmented rice brans and some isolated components has been proved *in vitro* [48]. *O. sativa* cooked seeds suppressed intestinal secretion through inhibiting the response of intestinal epithelial crypt cells to adenosine 3′,5′-cyclic monophosphate, a major intracellular mediator of secretion [51]. Rice fluid exhibited strong bactericidal activity against *H. pylori* [49]. In spite of these supportive data, Jayaraj et al. demonstrated that oil derived from rice and rice bran on storage becomes ulcerogenic, while fresh rice bran diet protected mucosa from ulceration [52]. The study evaluating dietary profile of patients with duodenal ulcer showed more ulcer occurrence in patient with rice diets. Moreover, mucin activity was attenuated, and severity of ulcer induced by pylorus ligation was higher in rice diet rats [53]. 

### 2.10. *Phoenix dactylifera* L.

Fruit and seed possess antioxidant activity [54, 55]. The fruit ameliorated gastric ulcers via increasing gastric mucin and reducing histamine and gastrin (a gastrointestinal hormone that regulates gastric acid secretion, releases histamine, and regulates gastric endocrine cell proliferation in the plasma) [56]. 

### 2.11. *Phyllanthus emblica* L.

*P. emblica* fruit purified phenolics demonstrated antioxidant activity *in vitro* [57]. The fruit exhibited wound healing activity via improvement of collagen function and enhancing antioxidant capacity [59]. It protected against gastric ulcer via its antioxidant and cytoprotective activity [60, 62]. Gallic acid enriched extract exhibited healing property on gastric ulcer via increasing PG E_2_ and proangiogenesis factors, enhancing endothelial NOS (eNOS), and regulation of pro-inflammatory and anti-inflammatory cytokines and antioxidant activity [61, 63]. The fruit ethanol extract demonstrated anti *H. pylori* activity *in vitro* [58].

### 2.12. *Punica granatum* L.

*P. granatum* peel extract protected gastric mucus from gastric ulcer via its antioxidant activity and attenuating gastric acidity [67, 68]. It attenuated acetylcholine-induced contractions and inhibition of the spontaneous movement of the isolated rat ileum [64]. The peel also showed anti *H. pylori* activity [65]. The ointment prepared from the peel extract accelerated wound healing and exhibited antioxidant properties in guinea pigs [66]. The tannins from fruit prevented formation of gastric ulcer, increased NO level and secretion of adherent and free mucus, and exhibited antioxidant activity in gastric mucosa [69].

### 2.13. *Rhus coriaria* L.

The fruit demonstrated antioxidant activity *in vitro* [71, 72]. Ethanol extract of fruit showed antibacterial activity against *H. pylori* [70].

### 2.14. *Vitis vinifera* L.

The seed demonstrated antioxidant activity *in vitro* [73]. The fruit skin and seed revealed anti *H. Pylori* effects [74, 75]. Proanthocyanidin-rich extract from seed protected against acute and chronic gastric and intestinal oxidative injury through inhibition of lipid peroxidation and membrane microviscosity in gastric and duodenal membrane [78]. It showed higher gastroprotective and antioxidant activity compared to vitamin E and C [80]. The seed also exhibited protective effect against gastric ulcer in rat. Antioxidant activity and strong ability of procyanidins to bind protein covering the stomach surface may be responsible for this protective affect [79]. This protein elevates defense activity of gastric membrane. The seed showed wound healing properties via enhancing angiogenesis and antioxidant activity [81, 83]. Resveratrol, a high abundant polyphenol in red grape fruits, suppressed *H. pylori* growth, *H. pylori*-induced interleukin-8 secretion, reactive oxygen species generation, and morphological changes in human gastric epithelial cells [76, 77]. Resveratrol in low dose (2 mg/Kg) demonstrated ulcer healing activity but in high dose (10 mg/Kg) was ulcerogenic. The mechanism of ulcer healing activity in low dose is attributed to inhibition of neutrophil aggregation, stimulation of COX1, PG E_2_, and eNOS, and improvement of angiogenesis [82]. 

## 3. Discussion

In TIM, a wide range of medicinal plants have been proposed for the treatment of different gastrointestinal disorders like inflammatory bowel disease, irritable bowel disease, hemorrhoids, and PU [84–87]. In this paper, all of edible fruits and spices claimed to be efficacious in the management of PU were collected from TIM sources, and any scientific evidence that prove their efficacy was retrieved from electronic databases. These remedies have shown their effectiveness on PU via several mechanisms of action including PG enhancement, modulation of inflammatory mediators, and antioxidant, anti *H. pylori*, wound healing, cytoprotective, and antisecretory activities. Some of the investigated fruits and spices like *Myristica fragrans*, *Phyllantus embelica*. *Vitis vinifera,* and *Punica granatum* have shown their beneficial effects in PU by affecting various associated mechanisms. According to published investigations, these fruits and spices seem to be more effective in the management of PU than the other ones. In contrast, for some of these fruits and spices including *Morus *species*, Cornus mas, Rhus cariaria, *and *Phoenix dactylifera*, just one or two studies on the efficacy and relevant mechanisms have been executed. Advanced scientific studies for evaluation of these herbs on PU and their possible mechanisms are suggested.

 Despite many pieces of *in vitro* and *in vivo* evidence, no human study was found to confirm the effectiveness of investigated fruits and spices in PU. As shown in Table 1, the plants used in TIM for management of PU are from different families, and there is no exact relationship between the family of plants investigated and their efficacy. No potential side effects have been reported from these remedies. Studies on antiulcer activity of some of investigated fruits and spices have revealed controversial results. For example, stored rice bran oil has shown ulcerogenic activity. Whereas, fresh rice bran diet and rice diet have demonstrated anti-PU properties in animal models [52, 53]. Fruit polyphenol extract of *Malus domestica* has ulcerogenic effect [27]. In contrast, fruit juice, flavonoids extract, and fruit methanol extract have shown gastroprotective activity in various animal models [30, 31, 34]. Despite different reports on protective activity of berberine, an active compound of *Berberis vulgaris*, against gastric ulcer [19, 20], there is a report about dose-dependent gastric ulcer inducing activity of this compound [21]. Some of the investigated remedies have shown conflicting results in different doses. Resveratrol, a highly abundant polyphenol in *Vitis vinifera* fruit, in low dose demonstrated ulcer healing activity but in high dose was ulcerogenic [82].

Overall, there are various edible fruits and spices in TIM for the management of PU which their efficacy had confirmed through various *in vitro* and *in vivo* studies. Because of the lack of human studies, it is recommended to conduct clinical trials to prove their efficacy and obtain more conclusive results. 

## Figures and Tables

**Table 1:** Medicinal plants used for the treatment of peptic ulcer in traditional Iranian medicine [8, 9].

Scientific names	Family	Common English name	Name(s) in TIM resources	Uses in TIM
*Amygdalus communis *	Rosaceae	Almond	Badam, Lowz	Respiratory disorders, brain tonic, and PU
*Berberis vulgaris *	Berberidaceae	Common barberry	Zereshk, Ambarbaris, and Arghis (root)	Gastric tonic, liver disease, dyspepsia, and PU
*Cornus mas *	Cornaceae	Cornelian cherry	Zoghal, Zoghal akhte	Gastritis, hepatitis, IBD, and PU
*Cucurbita maxima*,* C. pepo *	Cucurbitaceae	Pumpkin	Kadou, Ghar	Wound healer, and PU
*Cydonia vulgaris *	Rosaceae	Quince	Safarjal, Beh	Antidepressant, gastralgia, and PU
*Malus domestica *	Rosaceae	Apple	Sib, Toffah	Antidepressant, dysentery, and PU
*Morus alba*,* M. nigra *	Moraceae	White and black mulberry	Tout sefid, Tout siah	Liver and spleen disorders, aphrodisiac, diuretic, and PU
*Myristica fragrans *	Myristicaceae	Nutmeg, mace (aril of seed)	Jowz bouya (fruit), Basbase (aril of seed)	Gastric and liver tonic, PU, and aphrodisiac
*Oryza sativa *	Gramineae	Rice	Oroz, Berenj	IBD, PU, and aphrodisiac
*Phoenix dactylifera *	Arecaceae	Date	Khorma	Antidepressant, wound healer, aphthous, and PU
*Phyllanthus emblica *	Phyllanthaceae	Gooseberry	Amole	Memory enhancer, appetizer, and PU
*Punica granatum *	Punicaceae	Pomegranate	Anar, Roman	Gastric and liver tonic, PU, and IBD
*Rhus coriaria *	Anacardiaceae	Sumac	Sumac	Gastric tonic, appetizer, PU, and hemorrhage
*Vitis vinifera *	Vitaceae	Grape	Mow (tree), Ghoureh (unripe fruit), and Angour (ripe fruit)	Wound healer, hematopoietic

TIM: traditional Iranian medicine, PU: peptic ulcer, and IBD: inflammatory bowel disease.

**Table 2:** Pharmacological activities attributed to antipeptic ulcer activity of edible fruits and spices used in TIM for the management of this disease.

Plant	Part/extract	Active constituent	Model	Species	Result	Reference
*Amygdalus communis *	Powdered fruit	—	*In vitro*	—	Antacid	[10]
	Hull and shell/methanol extract	—	*In vitro*	—	Antioxidant	[11]
	Defatted seed/80% acetone extract and its fractions	—	*In vitro*	—	Antioxidant	[12]
	Nut/oil	—	Open wound	Rat	Wound healing	[13]
	—	Amygdalin	Ethanol-induced GU	Rat	↓GU, gastric secretion and inflammatory agents: TNF-*α* and NO	[14]

*Berberis vulgaris *	Fruit/ethanol, methanol and water extract	—	*In vitro*	—	Antioxidant	[15]
	—	Berberine	*In vitro*	—	↑NO in intestinal endothelium cell	[16]
	—	Berberine	*In vitro*	—	Anti-*H. pylori *	[17]
	Fruit/water extract	—	—	Broiler chicken	Improvement of intestinal mucosal morphology	[18]
	—	Berberine	Ethanol-induced GU	Mouse	↓GU, ↑eNOS, and ↓iNOS mRNA expressions	[19]
	—	Berberine	Indomethacin-induced small intestinal injury	Mouse	↓Intestinal injury, ↑adenosine of intestinal tissue	[20]
	—	Berberine	Acute toxicity	Mouse	Induction of GU	[21]

*Cornus mas *	Fruit/methanol extract	—	*In vitro*	—	Antioxidant	[22]

*Cornus controversa, C. macrophylla*, and* C. walteri *	Leaf/methanol extract	—	*In vitro*	—	Anti-*H. pylori *	[23]

*Cucurbita pepo *	Seed	Triterpenoids	*In vitro*	—	Antioxidant	[24]
	Ripe fruit pulp/aqueous extract	—	Aspirin-induced GU and DU	Rat	↓GU and DU, ↑mucosal thickness, and ↑alkaline phosphatase enzyme in stomach and duodenum tissue	[25]
	Seed	Triterpenoids	Pyloric ligation-, water immersion stress-, and indomethacin-induced GU	Rat	↓GU in all models, ↓gastric secretion, and ↓free and total acidity of gastric juice	[24]

*Cydonia vulgaris* syn. *C. oblonga *	Pulp, peel, and seed/methanol extracts	—	*In vitro*	—	Antioxidant	[26]
	Fruits/phenolic extract	—	*In vitro*	—	Antioxidant	[27]
	Fruits/phenolic extract	—	Ethanol-induced GU	Rat	↓GU	[27]
	Seed/mucilage	—	Toxin-induced skin lesions	Rabbit	Healing activity on toxin-induced lesion	[28]
	Fruits juice/70% ethanol extract	—	*In vitro*	—	Anti-*H. pylori* activity	[29]

*Malus domestica *	Fruit juice and flavonoids rich extract		*In vitro*	—	Antioxidant	[30]
	Fruit/methanol extract	Catechin and chlorogenic acid	*In vitro*	—	↓Gastric endothelial cell injury caused by xanthine-xanthine oxidase and indomethacin, ↑antioxidant activity, and ↓lipid peroxidation	[31]
	Fruit peel/polyphenol-rich extract	—	*In vitro*	—	Anti-*H. pylori*, inhibition of *H. pylori*-caused oxidant and free radical production, and bacterial toxin vacuolation and adhesion to tissues	[32]
	Fruit/juice and flavonoids extract	—	HCl/ethanol-induced GU	Rat	↓GU, ↓MPO activity in gastric tissue	[30]
	Fruit/methanol extract	—	Indomethacin-induced PU	Rat	↓PU, ↓lipid peroxidation and oxidative agents in gastric tissue	[31]
	Peel/polyphenol-rich extract	—	*H. pylori* infection	Mouse	Suppression of *H. pylori*-associated gastritis, inflammation and MDA levels in gastric tissue	[33]
	Fruit/polyphenol extract	—	Aspirin-induced and pylorus ligation-induced GU	Rat	↓GU in both models, no effect on gastric juice secretion, inhibition of aspirin-induced lipid peroxidation, and ↓COX2 and HB-EGF mRNA and protein over expression	[34]
	Fruit/polyphenol extract	—	Ethanol-induced GU	Rat	↑GU	[35]

*Morus alba *	—	Cyanidin-3-Oglucoside	*In vitro*	—	Improvement of endothelial dysfunction	[36]
	Leaf/water and 80% ethanol extracts	—	*In vitro*	—	Anti-*H. pylori*, antioxidant	[37]
	Fruit/ethyl acetate soluble fraction	—	Stress-induced GU	Rat	↓GU, ↓oxidative stress in tissue	[38]
	Leaf/ethanol extract	—	Ethanol-induced GU	Rat	↓GU	[35]

*Myristica fragrans *	Seed/methanol extract	—	*In vitro*	—	Anti-*H. pylori *	[39]
	Aril of seed	Dihydroguaiaretic acid	*In vitro*	—	Anti-*H. pylori *	[40]
	Aril of seed/acetone extract and its lignans rich fraction	Lignans	*In vitro*	—	Antioxidant	[41]
	Seed	—	Carbachol-induced gastric secretion	Rabbit	↓Gastric secretion, ↓free and total acidity of gastric juice	[42]
	Fruits in a polyherbal formulation	—	Pylorus ligation-induced GU	Rat	↓GU, suppression of gastric hypersecretion	[43]

*Myristica malabarica *	Fruit rind/methanol extract	—	Indomethacin-induced GU	Mouse	↑GU healing, ↑PGE2 synthesis, and ↑angiogenesis by ↑pro-angiogenics: VEGF and EGF	[44]
	Fruit rind/methanol extract	—	Indomethacin-induced GU	Mouse	↑Ulcer healing, ↑eNOS and ↓iNOS expressions, and balance between proinflammatory and anti-inflammatory cytokines	[45]
	Fruits rind/methanol extract	Procyanidins	Indomethacin-induced GU	Mouse	↑GU healing, ↑mucin content, and ↓lipid peroxidation and ↑antioxidant activity of gastric tissue	[46]

*Myristica andamanica *	Leaf/methanol extract	—	Excision wound	Mouse	Wound healing activity	[47]

*Oryza sativa *	Rice bran/methanol extract	Anthocyanins, *α*-tocopherol, and *γ*-oryzanol	*In vitro*	—	Antioxidant	[48]
	Fluid from unpolished and polished raw rice and popularly cooked Japanese rice	—	*In vitro*	—	Anti-*H. pylori *	[49]
	Bran oil	—	Stress-induced GU	Rat	↓GU, ↓stress-induced acid secretion, and ↓basal and stimulated acid secretion	[50]
	Cooked fruit	—	Intestinal secretion assay	Guinea pigs	↓Intestinal secretion, ↓response of intestinal crypt cells to cAMP	[51]
	Stored rice bran oil, fresh rice bran diet	—	Pylorus ligated ulcer	Rat	↑Ulcer by stored rice bran oil, ↓ulcer by fresh rice bran diet	[52]
	Rice diet	—	Pylorus ligated ulcer	Rat	↓Gastric secretion, ↓mucin activity, and ↑ulcer severity	[53]

*Phoenix dactylifera *	Fruit/methanol-water extract	—	*In vitro*	—	Antioxidant	[54]
	Seed/oil	—	*In vitro*	—	Antioxidant	[55]
	Fruit and pit/aqueous and ethanol extracts	—	Ethanol-induced GU	Rat	↓GU, ↑gastric mucin, ↓histamine in the gastric mucosa, and ↓gastrin in plasma	[56]

*Phyllanthus emblica *	Fruit	Phenolic compounds	*In vitro*	—	Antioxidant	[57]
	Fruit/ethanol extract	—	*In vitro*	—	Anti-*H. pylori *	[58]
	Fruit/ethanol extract	—	Excision wound	Rat	↑Wound healing, collagen function improvement, and ↑antioxidant enzymes: SOD, GSH, and GPx	[59]
	Fruit/polar solvent extract	—	Indomethacin-GU	Rat	↓GU, ↑antioxidant activity, and cytoprotective activity: ↑mucus and hexosamine	[60]
	Fruit/gallic acid enriched ethanol extract	—	Indomethacin-induced GU	Mouse	↑GU healing, ↑PGE2, and ↑proangiogenesis factors: VEGF, EGF, von Willebrand Factor VIII, and ↑eNOS/iNOS ratio	[61]
	Fruit/juice and methanol extract	—	Ethanol-, indomethacin-, and histamine-induced GU	Rat	↓GU in all models, ↓intraluminal bleeding, and ↑GSH of mucus	[62]
	Gallic acid enriched ethanol extract		Indomethacin-induced GU	Mouse	↑GU healing, proinflammatory and anti-inflammatory cytokines regulation, antioxidant activity, and ↓lipid peroxidation	[63]

*Punica granatum *	Fruit peel/aqueous extract	—	*In vitro*	—	↓Acetylcholine-induced contractions, ↓spontaneous movement of the isolated rat ileum	[64]
	Fruit peel/methanol extract	—	*In vitro*	—	Anti-*H. pylori *	[65]
	Fruit peel/methanol extract	—	Excision wound	Guinea pig	↑Wound healing, ↑collagen, DNA, and tissue proteins	[66]
	Fruit peel/aqueous extract	—	Ethanol-induced GU	Rat	↓GU, ↓gastric acidity	[67]
	Fruit peel/methanol extract	—	Aspirin- and ethanol-induced GU	Rat	↓GU in both models, ↑catalase, ↑GSH, ↑GPx, ↑SOD, and ↓lipid peroxidation	[68]
	Fruit	Tannins	Water immersion stress-, pylorus ligation-, and intragastric absolute ethanol-induced ulcer	Rat	↓Lipid peroxidation, ↑NO, ↑GPx, ↑SOD in gastric mucosa, and ↑secretion of adherent mucus and free mucus	[69]

*Rhus coriaria *	Fruit/ethanol extract	—	*In vitro*	—	Anti-*H. pylori *	[70]
	Fruit/aqueous extract	Gallic acid	*In vitro*	—	Antioxidant activity, ↓oxidative stress, and ↓lipid peroxidation in rat isolated hepatocytes	[71]
	Fruit/methanol extract	—	*In vitro*	—	↓lipid peroxidation, Antioxidant activity	[72]

*Vitis vinifera *	Seed/various extract	—	*In vitro*	—	Antioxidant	[73]
	Fruit skin and seed/various extract	—	*In vitro*	—	Anti-*H. pylori *	[74]
	Fruit/hydroalcoholic extract	—	*In vitro*	—	Anti-*H. pylori *	[75]
	Fruit juice	Resveratrol	*In vitro*	—	Anti-*H. pylori *	[76]
	Fruit juice	Resveratrol	*In vitro*	—	Anti-*H. pylori*, ↓ROS, ↓inflammatory agents, and improvement of gastric mucosal cell morphological changes induced by *H. pylori *	[77]
	Seed/proanthocyanidin extract	Resveratrol	Acute and chronic water-immersion restraint stress-induced gastric and intestinal oxidative injury	Rat	↓GU and DU, ↓lipid peroxidation, and ↓gastric and duodenal membrane microviscosity	[78]
	Seed/low and high flavanol content extract, procyanidins extract	—	Ethanol/HCl-induced GU	Rat	↓GU, radical scavenging activity, and procyanidins binding ability to stomach surface protein which result in ↑defense activity of gastric membrane	[79]
	Seed/proanthocyanidin rich extract	—	Aspirin- and ethanol-induced GU	Rat	↓Ulcer in both models, ↓lipid peroxidation more than Vit E and Vit C	[80]
	Seed/proanthocyanidin extract	—	Excision wound	Mouse	↑Wound healing, ↑angiogenesis activity and factor: VEGF, and ↑antioxidant function of tissue	[81]
	—	Resveratrol	Aspirin-induced GU	Rat	2 mg/Kg: ↑GU healing, ↓MPO, ↑COX1, ↑PGE2, ↑eNOS, and ↑angiogenesis; 10 mg/Kg: ulcerogenic	[82]

cAMP: adenosine 3′5′-cyclic monophosphate; COX: cyclooxygenase; DU: duodenal ulcer; EGF: epidermal growth factor; eNOS: endothelial NO synthase; GPx: glutathione peroxidase; GSH: glutathione; GU: gastric ulcer; *H. pylori*: *Helicobacter pylori*; HB-EGF: heparin-binding EGF-like growth factor; iNOS: inducible NO synthase; MDA: malondialdehyde; MPO: myeloperoxidase; NO: nitric oxide; PGE2: prostaglandin E2; PU: peptic ulcer; ROS: reactive oxygen species; SOD: superoxide dismutase; TNF-*α*: tumor necrosis factor-alpha; and VEGF: vascular EGF.

## Abbreviations

COX: Cyclooxygenase
eNOS: Endothelial nitric oxide synthase
GSH: Glutathione
*H. pylori*: *Helicobacter pylori*
HB-EGF: Heparin-binding epidermal growth factor-like growth factor
NO: Nitric oxide
NOS: Nitric oxide synthase
NSAIDs: Non steroidal anti-inflammatory drugs
PU: Peptic ulcer
TIM: Traditional Iranian medicine
TNF-*α*: Tumor necrosis factor-alpha.
